# Acute lymphoblastic B‐cell leukemia or CAR‐T cell related skin changes – a relevant diagnostic challenge

**DOI:** 10.1111/ddg.15931

**Published:** 2025-10-06

**Authors:** Farzan Solimani, Amrei Dilling, Konrad Heisterkamp, Frederik Damm, Olaf Penack, Christian Oberender, Philipp Le‐Coutre, Martina Rudelius, Kamran Ghoreschi, Alexander Nast

**Affiliations:** ^1^ Department of Dermatology Venereology and Allergology Charité – Universitätsmedizin Berlin corporate member of Freie Universität Berlin Humboldt‐Universität zu Berlin and Berlin Institute of Health Berlin Germany; ^2^ Department of Hematology Oncology and Tumorimmunology Charité – Universitätsmedizin Berlin corporate member of Freie Universität Berlin and Humboldt‐Universität zu Berlin Campus Virchow Klinikum Berlin Germany; ^3^ Department of Hematology Oncology and Tumorimmunology Charité – Universitätsmedizin Berlin corporate member of Freie Universität Berlin and Humboldt‐Universität zu Berlin Campus Charité Mitte Berlin Germany; ^4^ Institute of Pathology Ludwig‐Maximilians‐University of Munich Munich Germany

Dear Editors,

Chimeric antigen receptor (CAR) therapy is bringing revolutionary therapeutic concepts into clinical practice and enabling physicians to deplete specific cells.^1^ CAR‐T cells are based on genetically engineered T cells that selectively target specific antigens.^1^ The increasing numbers of patients receiving CAR‐T therapy is also leading clinicians to encounter rare adverse events more frequently. Cutaneous adverse events of CAR‐T therapy are poorly understood and rarely described and can challenge clinicians to differentiate between recurrence of disease and secondary cutaneous toxicity. A recent case from our department illustrates the clinical and diagnostic challenges of CAR‐T cell induced cutaneous adverse events.

A 50‐year‐old man was diagnosed with a *BCR‐ABL* and *KMT2A* negative acute lymphoblastic B‐cell leukemia (B‐ALL) and initially treated with a chemotherapeutic induction regimen (dexamethasone, cyclophosphamide, daunorubicin, peg‐asparaginase, cytarabine) and rituximab. This triggered an antithrombin III deficiency, and, in accordance with the patient's wishes, the treatment was terminated. Eight months later the patient presented to our outpatient dermatological clinic due to the occurrence of infiltrated plaques on the occipital side of head. A histological examination showed nodular partly diffuse blastic infiltrate with CD19^+^, PAX5^+^, CD3^‐^ profile and a high (80%) Ki67 proliferation index (Figure 1a). A recurrence of B‐ALL was diagnosed. Eighteen months later salvage therapy with blinatumomab (a bispecific monoclonal antibody (mAB) T‐cell engager that recognizes and links CD3 and CD19) was started but was dismissed shortly thereafter due to generalized serositis. After a thorough explanation of the available options, the patient declined an allogeneic transplantation and opted for anti‐CD19 CAR‐T treatment, which was started 4 months later. After bridging therapy with inotuzumab ozogamicin (a humanized mAb against the B‐cell marker CD22 linked to a cytotoxic agent) and conditioning (fludarabine and cyclophosphamide) the patient received 1 x 10^6^‐CAR‐T cells/kg (brexucabtagene autoleucel, an autologous CD19 directed CAR). A grade II cytokine release syndrome (CRS) was promptly managed with tocilizumab (an anti‐IL‐6 receptor mAb). Eight months after CAR‐T infusion the patient presented in our department again due to the onset of painless non‐pruriginous macules and slightly infiltrated plaques and nodules on the occipital side of the head, abdominal region and knee (Figure 2). Additionally, the patient reported to have morning stiffness, joint pain and myalgia. There was no evidence of any further organ‐related toxicity. The medical history also ruled out the possibility of a contact allergic reaction, viral exanthema or vaccine‐related reaction. Apart from a slight decrease in circulating neutrophils and an increase in monocytes, the differential blood count showed no pathological changes in the circulating CD4/CD8 T and natural killer (NK) cells. We suspected a recurrence of B‐ALL and performed histological examination of skin biopsy from the abdominal regional and occipital region. Histology revealed a dense lymphocytic and eosinophilic infiltration with perivascular accentuation. The lymphocytic infiltration was made up almost exclusively by a small mixed CD4/CD8 CD30^–^ T cells (Figure 1b–f), whereas B cell‐lineage specific markers (CD20, CD19, Pax5) showed only a few positive cells, thus excluding the presence of a B‐cell malignancy (Figure 1g–h). Ki67 was also not significantly elevated and terminal deoxynucleotidyl transferase (TdT, a marker for lymphoblastic lymphomas and leukemias) was negative (Figure 2). A clonality assessment of the immunoglobulin heavy chain (IgH, framework regions FR1‐4) and of the T‐cell receptor (TCR)γ (Vγ/J, Vγ/Jp) showed a polyclonal IgH repertoire and absence of TCR‐clonality. These results pointed against the presence of B‐ALL or other haemato‐oncological diseases and we made a diagnosis of CAR‐T cell induced T‐cell pseudo lymphomatous skin reaction. The lesions improved rapidly in the first 3 months, after which they showed some symptom‐free residual inflammation that was resistant to treatment. Twelve months after CAR‐T cell treatment, the patient is still in remission at follow‐up examinations with regard to B‐ALL and still shows slight residual erythematous lesions.

**FIGURE 1:**
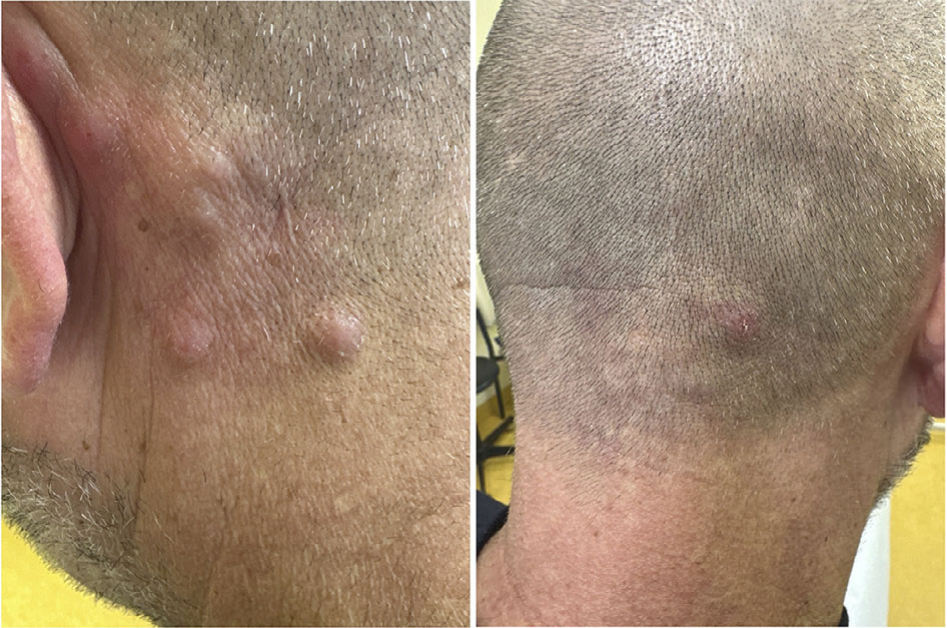
(a) Clinical manifestations of CAR‐T cell–induced cutaneous changes in a 50‐year‐old male patient treated with anti‐CD19 CAR‐T cells for acute lymphoblastic leukemia.

**FIGURE 2:**
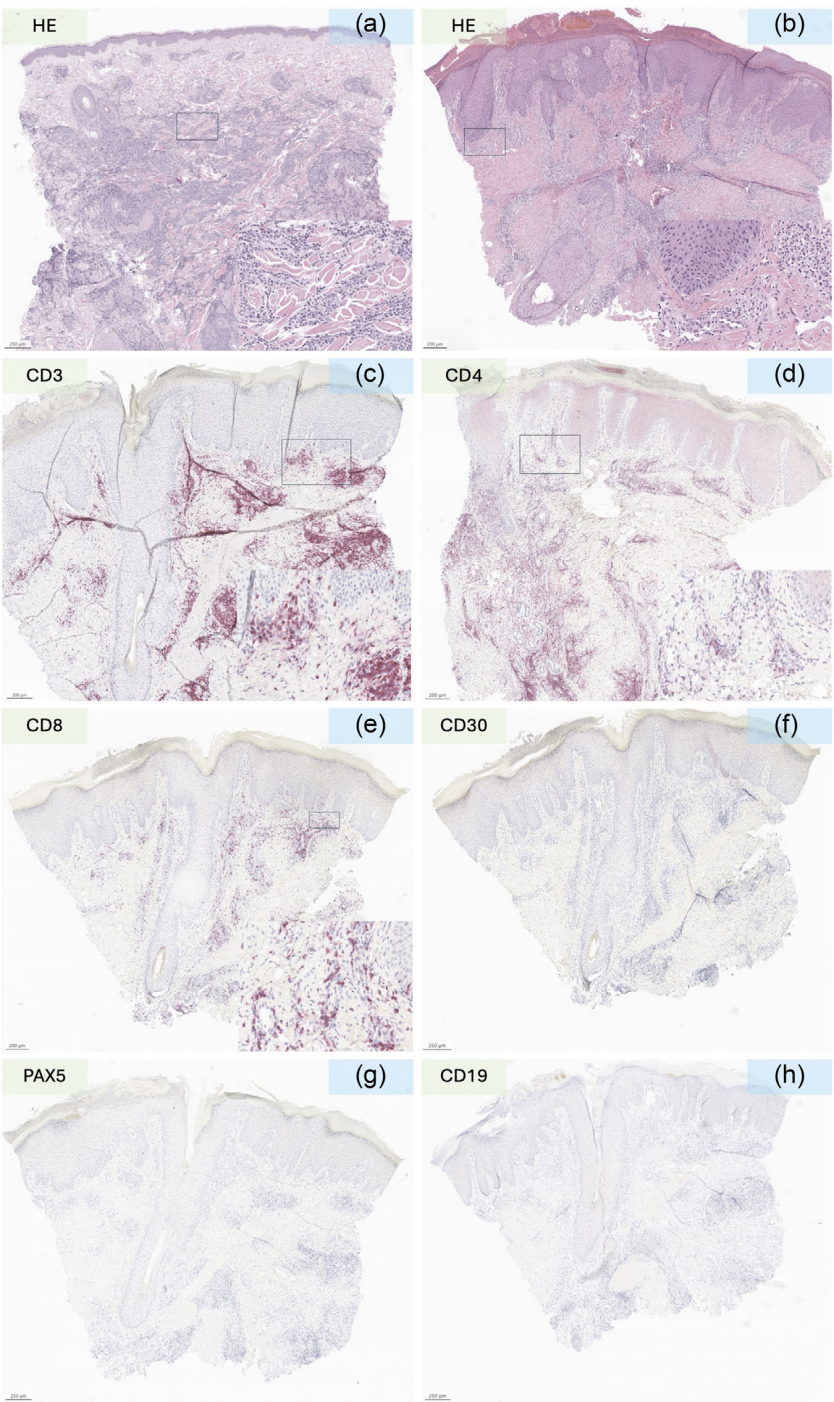
(a) Hematoxylin and eosin (HE) staining of acute lymphoblastic B‐cell leukemia (B‐ALL)‐induced leukemia cutis before treatment with anti‐CD19 CAR‐T cells; (b) HE staining of anti‐CD19 CAR‐T cell–induced cutaneous side effect; (c–f) T‐cell–specific staining (CD3, CD4, CD8, CD30) showing a mixed CD30‐negative CD4/CD8 lymphocytic infiltrate; (g, h) PAX5 and CD19 staining showing only sparse B cells infiltrating the affected skin. Scale bar = 250 µm (a, f–h) or 200 µm (b–e), as indicated in the lower left corner of each staining. (a–e) Details of dermal cellular infiltrates in the lower right boxes; scale bar = 20 µm.

While CRS and Immune effector cell‐associated neurotoxicity syndrome (ICANS) are well described adverse events, cutaneous manifestations need to be elucidated and poses a clinical challenge, especially since it might mimic leukemia cutis. Reports described so far different cutaneous manifestation such as maculopapular eruptions, erythematous rashes, purpura, petechiae, and blistering lesions.^2^, ^3^ A recent study on CAR‐T clinical trials showed that mild‐to‐moderate skin reactions occur in up to 35% of enrolled patients.^2^ Lack of dermatological training could conceal higher rates. For physicians, it is crucial to thoroughly exclude a recurrence of disease. We performed immunohistochemistry and clonality assessment to rule out leukemia cutis. Other groups took different approaches. One group applied flow cytometry of blister fluids and skin‐lesional T cells from a patient with bullous eruption, which showed presence of CD3 mixed CAR‐T^+^/CAR‐T^–^ cells.^4^ In a study involving CD30 CAR‐T cell therapy in Hodgkin lymphoma skin reactions were analyzed by histology and quantitative polymerase chain reaction showing expression of the CD30 CAR transgene.^5^ The underlying immunopathomechanism for CAR‐T‐induced skin reactions still needs to be clarified. In our patient, the skin reaction interestingly occurred in the occipital region, where the patient originally developed leukemia cutis. This might indicate a possible initial reaction to malignant resident B‐cells as *primum movens* for skin infiltration. Concordantly to this concept, Hagen et al. recently described a new form of late‐onset CAR‐T‐related organ toxicity (local immune effector cell‐associated toxicity syndrome (LICATS)) in patients with autoimmune diseases who developed inflammatory reactions in the affected organs due to CAR‐T‐mediated B‐cell killing. The reported skin lesions are very similar to those observed in our patient, which also occurred as a result of B‐cell killing in the skin^6^.

## CONFLICT OF INTEREST STATEMENT

None.
